# Effects of Electrode Materials and Compositions on the Resistance Behavior of Dielectric Elastomer Transducers

**DOI:** 10.3390/polym15020310

**Published:** 2023-01-07

**Authors:** Tobias Pascal Willian, Bettina Fasolt, Paul Motzki, Gianluca Rizzello, Stefan Seelecke

**Affiliations:** 1 Department of Systems Engineering, Saarland University, 66123 Saarbrücken, Germany; 2 Center for Mechatronics and Automation Technologies (ZeMA) gGmbH, 66121 Saarbrücken, Germany; 3 Department of Materials Science and Engineering, Saarland University, 66123 Saarbrücken, Germany

**Keywords:** dielectric elastomer, material characterization, carbon black, PDMS, electrical resistance, flexible electrodes

## Abstract

Dielectric elastomer (DE) transducers possess various advantages in comparison to alternative actuator technologies, such as, e.g., electromagnetic drive systems. DE can achieve large deformations, high driving frequencies, and are energy efficient. DEs consist of a dielectric membrane sandwiched between conductive electrodes. Electrodes are especially important for performance, as they must maintain high electrical conductivity while being subjected to large stretches. Low electrical resistances allow faster actuation frequencies. Additionally, a rate-independent, monotonic, and hysteresis-free resistance behavior over large elongations enables DEs to be used as resistive deformation sensors, in contrast to the conventional capacitive ones. This paper presents a systematic study on various electrode compositions consisting of different polydimethylsiloxane (PDMS) and nano-scaled carbon blacks (CB). The experiments show that the electrode resistance depends on the weight ratio of CB to PDMS, and the type of CB used. At low ratios, a high electrical resistance accompanied by a bimodal behavior in the resistance time evolution was observed, when stretching the electrodes cyclic in a triangular manner. This phenomenon decreases with increasing CB ratio. The type of PDMS also influences the resistance characteristics during elongation. Finally, a physical model of the observed phenomenon is presented.

## 1. Introduction

**Figure 1:**
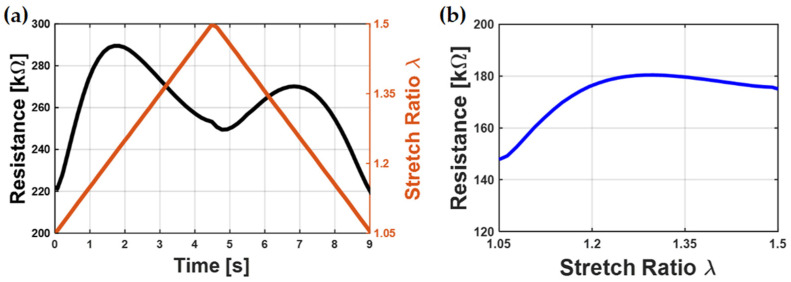
Typical bimodal behavior of DE resistance during stretching: (**a**) The electrical resistance and the stretch values over time, where the electrical resistance shows a bimodal behavior; (**b**) Electrical resistance over stretch: The resistance first increases but decreases again after reaching a maximum.

Research on DE transducers has grown rapidly over the past decades. As smart material systems, DEs can be used as actuators, sensors, or generators. Their applications include valves [1], pressure sensors [2], positioning systems [3], soft robots [4,5,6,7,8,9,10], smart wearables [11], and soft grippers [12], to mention a few. Other than novel applications, basic research concerning the material properties has also been the object of extensive study, e.g., [13,14,15,16,17,18]. Knowing and understanding how DE material behaves under different conditions is an important factor to push this technology forward. DEs consist of mainly two parts: an insulating dielectric material, and conductive electrodes which are applied onto the dielectric film. A variety of dielectric materials can be used, e.g., polydimethylsiloxane [10,19,20,21,22,23,24], acrylic materials such as VHB (“very high bond”, a group of materials from 3M, including different acrylic polymers) from 3M [17,25,26], or polyurethane [27]. The electrode often consist of polymers filled with conductive nano-particles such as carbon black (CB) [9,10,11,13,14,15,16,17,18,19,20,21,22,23,24,26,28], but other approaches including conductive polymers (e.g., poly(3,4-ethylenedioxythiophene)-poly(styrenesulfonate)(PEDOT:PSS)), metallic electrodes [29,30], and carbon fibers or carbon nano tubes [9,27,31,32] are also possible. Depending on the application, different properties are required of the DE dielectric membrane, e.g., high breakdown strength, high electrical permittivity, and homogeneous thickness. Additionally, the electrode properties must be adjusted to a specific application, e.g., good adhesion to the dielectric membrane, a high electrical conductivity, and a low thickness should be achieved and retained even when the DE is stretched. This paper focuses on the electrical resistance of CB-filled PDMS-based electrodes, and its behavior under elongation. The electrical resistance is a crucial factor for DEs. In high-frequency applications, a low resistance is required, as it allows fast electrical loading and unloading cycles. To use the electrical resistance as an alternative sensor signal for uniquely reconstructing the DE elongation, a hysteresis-free and monotonic resistance behavior is needed. It needs also to stay in a suitable range for the application even under high elongations. Understanding and knowing the resistance behavior could also allow new applications, such as monitoring electrode degradation by means of the change in resistance in long-term applications. Looking at earlier research, where the electrical resistance of filled polymeric conductive materials is investigated during elongation, the electrical resistance behaves in significantly different ways for different compositions: While many compositions exhibit a monotonically increasing electrical resistance with increasing elongation [27,33,34,35,36], the electrical resistance of other electrode materials shows a different (i.e., bimodal) behavior. An example of such behavior is shown in Figure 1.

First, as expected, the electrical resistance increases with increasing elongation. However, at a certain point, the electrical resistance starts to decrease, even though the stretch ratio still increases. When the maximal stretch is reached and the sample is released, the behavior is mirrored: the resistance increases first to a second maximum and then drops to its initial value when the sample is relaxed. This phenomenon could be seen for both displacement- and pressure-controlled testing conditions [7,8,24,27,33,37]. To understand and be able to control this behavior, this paper investigates the DE electrode resistance in relation to its material composition. CB-filled PDMS electrodes are used and manufactured via screen-printing. The screen-printing method is chosen, because it allows a fast and easily up-scalable process for future mass production manufacturing while additionally allowing a wide viscosity spectrum of processable electrode materials. Electrodes manufactured with this method yield a high stretchability while retaining electrical conductivity even under large elongations. By using this method, DEs with high quality reproducible properties are manufactured, and the results of this study can directly be transferred to future applications. Therefore, the investigation of the electrical resistance behavior focusses on electrodes manufactured with the screen-printing method.

The resistance behavior of the manufactured electrodes is not only influenced by the manufacturing process, but most importantly by their material composition, see [13,23,24,28], where the conductive filler ratio is reported to be an important factor. The quantity must be high enough to overcome the percolation threshold, and ensure high electrical conductivity [28,37,38,39], leading, in turn, to low electrical resistances. Therefore, investigating the behavior of different electrode compositions and their resistance characteristics is of great importance for the performance of DE actuators and DE sensors. This paper presents a comprehensive study on the resistance behavior of DEs during elongation. Investigated electrode material compositions vary in the use of CB type and concentrations as well as PDMS types and concentrations. The influence of both the PDMS matrix material and the CB filler is investigated. The first part focuses on different CBs and different CB to PDMS ratios to highlight the importance of the CB content, whereby all these samples have the same silicone as matrix material. In the second part of the study, different types of silicones are investigated. In this part, the CB to silicone ratio as well as the type of CB used is kept constant to exclude the influence of different CBs while focusing on the influence of the used silicone matrix. After discussing the results of the experimental campaign, a physical explanation based on the observed influences from the different material compositions explaining the observed phenomena will be presented in this paper. 

This work gives an understanding and explanation of the influences of the electrode composition on the electrical resistance behavior. This is especially important for the application of resistive DE sensors and high frequency actuators. As all DE electrodes investigated in this study are manufactured by screen-printing, the materials are additionally evaluated in respect of their screen-printability. The paper aims to give the reader a fundamental understanding on what to consider when designing DE-electrode-systems with strong reference to the electrical resistance.

## 2. Materials and Methods

### 2.1. Materials

WACKER ELASTOSIL 2030 ^®^ PDMS film with a thickness of 50 µm is used in this study as dielectric material. It is commercially available and widely used in studies and applications [10,19,20,21,22]. The materials used for manufacturing the DE electrodes are all commercially available and are listed in Table 1. On one hand, three commercially available silicones are chosen as matrix material. They are characterized by their stiffening effect, and their influence on the force–displacement hysteresis of the DEs while elongated (see Section 3.2). On the other hand, three different conductive nano-scaled carbon blacks differing in BET surface areas (Table 2) are chosen. BET-measurement is a method to investigate the surface area of solids, while BET is an abbreviation of the names of the developers of this method: S. Brunauer, P. H. Emmett, and E. Teller. Depending on this value, CBs can be classified as supra-conductive, extra-conductive, or ultra-conductive [40]. CBs with higher BET surface area are often more likely to build complex structures, and therefore result in higher electrical conductivities, and lower electrical resistances, respectively [39,41,42]. In addition, CB with different particle sizes, CB structures, and the interaction between filler particles and the matrix material influences the electrical resistance [19,35,43]. 

### 2.2. Sample Preparation

**Figure 2:**
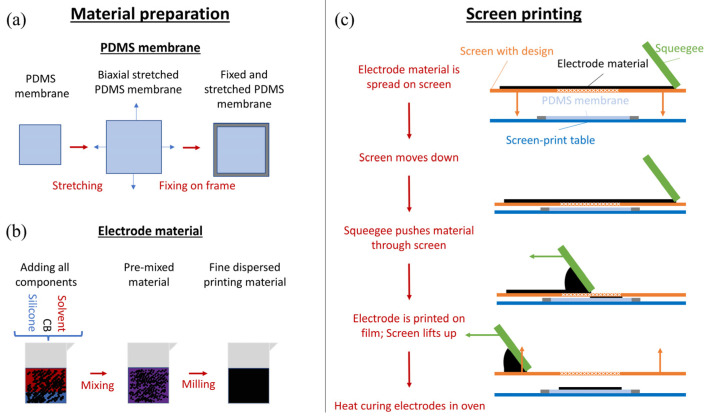
Schematic electrode manufacturing procedure: (**a**) Preparation of the silicone membrane. A piece of silicone membrane is stretched biaxially and then fixed between two metal frames, so that the center of the silicone film is not covered by the metal; (**b**) Preparation of electrode material. First, all components of the electrode ink are added into a cup, pre-mixed with a Thinky planetary mixer and subsequently milled with a three-roll mill to homogenize the printing ink; (**c**) Steps for screen-printing electrodes. The printing ink is spread over the screen with the desired design. The screen moves close to the silicone membrane. The squeegees move with a specific printing pressure over the screen and squeezes the material through the screen onto the silicone membrane. Subsequently, the screen lifts up again and the frame is transferred into the oven for curing.

This section provides a brief overview on the DE preparation process. It is divided into five steps:

1. Elastosil 2030 silicone film with a thickness of 50 µm is transferred to metal frames. In this study, the films are pre-stretched by 5% biaxially. The steps for preparing the dielectric membrane are shown in Figure 2a. The pre-stretch improves the handling of the film and allows an easier and more homogenous preparation of dielectric elastomers. In addition, pre-stretching the film increases the breakdown strength of the silicone membrane [44]. Pre-stretching the film also decreases its thickness, and therefore a lower voltage can be applied to reach the same electrical fields. These are reasons why pre-stretching the dielectric membrane is commonly performed. In this paper, no electro-mechanical measurements are performed, but the pre-stretching is still carried out due to the better handling of the membranes.
2. Preparation of electrode material: CB and silicone are mixed with solvent (VD60 from SunChemical, Parsippany, NJ, USA) and homogenized in a three-roll mill from EXAKT (Norderstedt, Germany) and a Thinky (Laguna Hills, CA, USA) planetary mixer to provide screen-printability (see Figure 2b). To adjust the viscosity of the screen-print material, and thus ensure the screen-printability of the samples, solvent is added to the mixture. More solvent is needed if the CB to silicone ratio increases, otherwise the printing material becomes too dry. While screen-printing, a specific amount of electrode material is squeezed through the screen, independent of the used material. If there is proportionally more solvent in the printed electrode composition, more of the material will vaporize during curing, therefore leading to a thinner electrode. If the amount of solvent required to enable screen-printability is too high, matrix material and CB filler will separate during the printing process and the mixture is not homogeneous anymore. This leads potentially to more imperfections in the printed electrodes. Such electrodes are not usable for the measurements conducted in this work. The prepared electrode compositions (CB to silicone ratios) and the corresponding amount of solvent needed to provide screen-printability are discussed in detail in Section 2.4. The mixtures containing too much solvent to yield a homogeneous print image are also reported in that section.
3. The electrodes are screen-printed on one side of the silicone film, using a SEFAR (Heiden, Switzerland) 90/48Y screen (90 threads per centimeter and a thread thickness of 48 µm) with a polyethylenterephthalate (PET) mesh. The influence of screen print parameters on the electrodes for DEs was previously investigated in [34]. Using this study, a screen was selected with a mesh size in between the very fine and medium coarse mesh of the study, yielding a higher ink throughput and clear print edges. This screen is a standard low-cost screen size. One electrode layer is printed, which is the standard procedure for screen-printing. A schematic screen-printing procedure is shown in Figure 2c.
4. After screen-printing, the electrodes are cured for one hour at 150 °C.
5. Finally, two monolithic 3D-printed reinforcement frames are applied to sandwich the film. These frames allow exact repeatability of the sample placement in the test rig.

### 2.3. Sample Geometry

**Figure 3:**
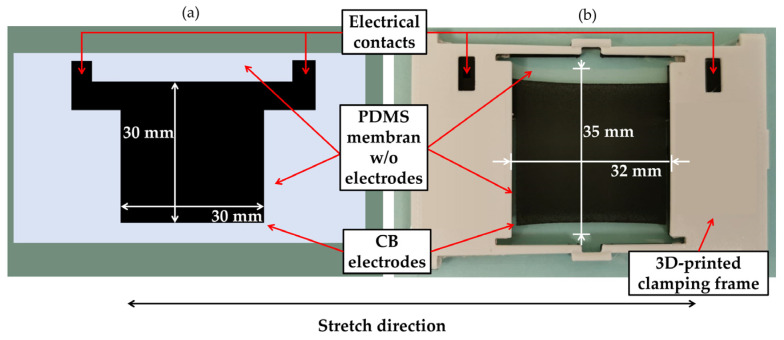
(**a**) Schematic sample geometry: 30 × 30 mm^2^ active electrode area printed on PDMS membrane. Arms are printed on both sides for electrical connection to the test rig. Arms are clamped in between a 3D-printed clamping frame and are not stretched during the measurements.; (**b**) actual sample inside 3D-printed clamp for testing in the universal test rig. The overall sample geometry is 32 mm × 35 mm, as there are 2.5 mm unprinted PDMS membrane on the free sides (top and bottom) and 1 mm unprinted PDMS membrane on both sides between the active electrode area and 3D-printed clamping frame.

Figure 3a shows schematically the electrode geometry and a picture of a manufactured sample used for the tests in this study can be seen in Figure 3b. The electrodes geometry is a square with an edge length of 30 mm, resulting in an electrode area of 30 mm × 30 mm. On the free edges, there are 2.5 mm unprinted silicone film, while the electrode and the 3D-printed frame are distanced by 1 mm. In total, the size of the resulting sample geometry equals 32 mm × 35 mm (length × width). In addition, two arms printed out of the electrode are added, to be used as an electrical contact with a measurement device (e.g., a LCR meter). Tensile tests are conducted in a custom-developed test rig. A detailed explanation and validation of the test rig can be found in [36].

### 2.4. Experiments

The experimental part, discussed in the next section, is divided into two sub-sections. For both sections, the electrodes are manufactured via the screen-printing method described in Section 2.2. The electrical resistance of the electrodes is measured with an LCR meter (HAMEG HM 8118, Rohde & Schwarz, Munich, Germany), set to a measuring frequency of 1 kHz. 

Section 3.1 focusses on the influence of CB type and ratio. To investigate this parameter, the silicone Silgel 612 A/B is used as matrix material for all manufactured samples in this section. Only changes induced by different CBs and changing CB to silicone ratios are observed. The weight ratio of CB is investigated in 5 wt% (wt% = weight percent) increments, starting from 5 wt% and increased stepwise up to 40 wt%. The range from 5 wt% to 40 wt% is chosen as it allows an investigation from a very low CB ratio (5 wt%), which is close to the percolation threshold for some CBs, to higher CB ratios, where the screen-printability is not given anymore. The screen-printing process limits the amount of CB in the printing material, as CB ratios which go beyond 40 wt% are not screen-printable anymore. The step size enables a detailed investigation of the influence of different CB to PDMS ratios. An overview of the manufactured electrode compositions for the first experimental section is shown in Table 3. In addition, the amount of solvent for the specific CB to PDMS ratios is shown, while it is chosen so that a smooth paste with a screen-printable viscosity is generated. The amount of solvent increases with increasing BET-surface area of the CB particle (BET-surface area of Ketjenblack EC 600-JD >> Ensaco 350G >> Vulcan XC-72, see Table 2). For some mixtures with very low or very high CB ratios, resistance measurements could not be conducted. If the CB ratios are too low (5 wt%), no electrical conductivity could be achieved, as the percolation threshold is not reached. If the CB ratio is too high and too much solvent is required, samples with a high BET surface area are not screen-printable or show a very inhomogeneous print image. This is, on one hand, due to the higher amount of solvent in the screen-print material and thus a lower electrode quality (as already stated in Section 2.2) and on the other hand, if the CB ratio increases in the electrode, the amount of silicone decreases. If the CB ratio becomes too high, the amount of silicone is not enough anymore to hold the electrode to the silicone film, resulting in a bad adhesion. In addition, too low PDMS ratios lead to a worse cohesion of the electrode, as the silicone is not enough to bind the huge amount of CB particles anymore. In Table 3, the material compositions which showed no measurable electrical resistance are marked with ‘n.e.c.’ (no electrical conductivity). Electrode compositions which are not screen-printable or showed a too poor electrode quality are marked as ′n.s.p.′ (not screen-printable). 

In comparison to Section 3.1, in which a fixed PDMS material is used to investigate the influence of different CB types and CB to PDMS ratios, Section 3.2 focusses on the influence of the silicone matrix material on the electrical resistance. Three different silicone matrix materials are investigated (see Table 1). The CB material and ratio are kept constant during all experiments conducted in Section 3.2. The electrode material is prepared using a mixture of 84 wt% silicone and 16 wt% CB. For the measurements conducted in this part, a CB ratio which differs from the ones used in Section 3.1 is used. Desired is an electrode with as few imperfections as possible to keep the influence of the silicones unaltered. Therefore, the electrodes must be of high quality. A mixture of 8 wt% Ensaco 350G and 8 wt% Ketjenblack EC-600JD in 84 wt% silicone has turned out to be favorable to manufacture the electrodes examined in this section, as they provide a very good screen-printability, and being with 16 wt% CB in a range far from too low or too high CB ratios. Additionally, because the CB ratio and material are constant in all experiments, differences in the electrical resistance behavior in the samples can only be attributed to the varied matrix materials. 

**Figure 4:**
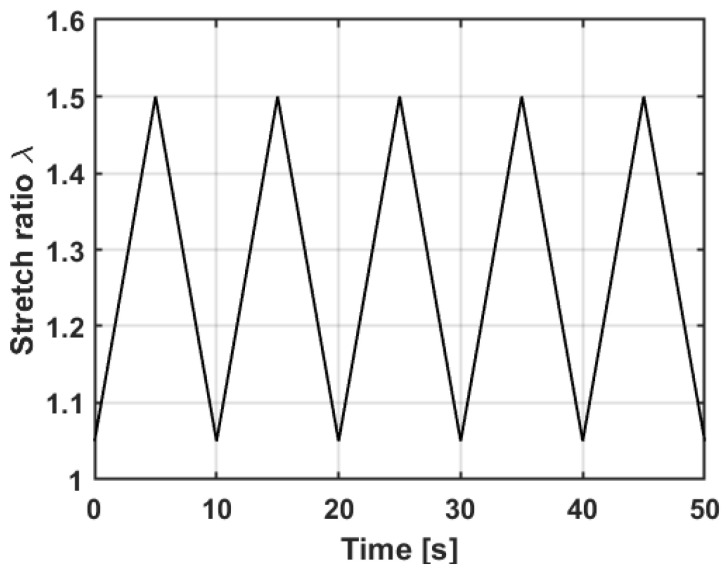
Specified stretch over time curve used for tests in this work: ε = 5–50% and the measuring frequency f = 0.1 Hz.

For both experimental sub-sections, samples are stretched in a linear and cyclic manner (compare to Figure 4). The measuring frequency for the elongation is chosen to be 0.1 Hz. To ensure the repetitive behavior of DEs during multiple strain cycles, the DEs are preliminarily trained. To this end, the samples are stretched six times before the actual measurement is carried out. Due to this training, reversible and irreversible effects occurring in the first few stretches (e.g., energy elastic effects or the Mullins effect [45]) are minimized. The experiments are performed immediately after the training, so that no long-term relaxation processes could occur. 

## 3. Results and Discussion

This section shows and discusses the experimental results, with the aim to classify the observed bimodal behavior of the resistance when stretching DEs. As stated above, Section 3.1 focusses on the influence of CB, while Section 3.2 shows the influence of different PDMS used as matrix materials. 

### 3.1. Influence of Carbon Black

**Figure 5:**
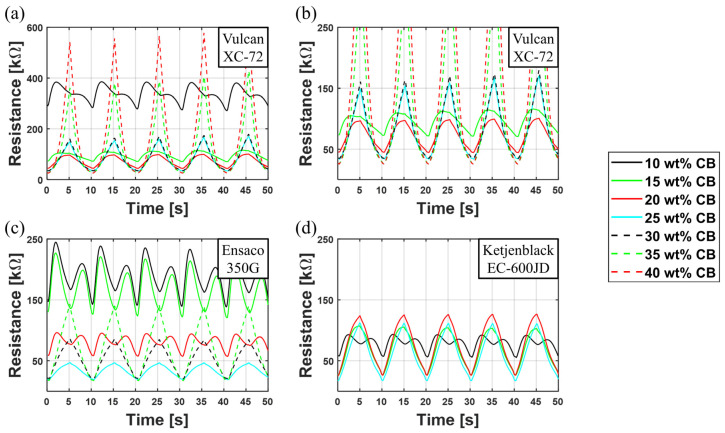
Influence of the used CB and the CB to silicone ratio on the electrical resistance: (**a**,**b**) resistance over time with different scaling for Vulcan XC-72; (**c**) resistance over time for Ensaco 350G (**d**) resistance over time for Ketjenblack EC-600JD.

Here, the influence of the CB type and ratio on the bimodal behavior is investigated. Figure 5 shows the resistance over time curves for all three carbon blacks. Vulcan XC-72 shows very high resistances for 10 wt%, 35 wt%, and 40 wt%, (Figure 5a) in comparison to Ensaco350G and Ketjenblack EC-600JD (Figure 5c and Figure 5d, respectively). For better comparison with the other CB mixtures, Figure 5b shows an expanded view of the Figure, by adopting the same resistance scale as in Figure 5c,d. For the lowest investigated CB ratio of 5 wt%, no electrical conductivity is achieved for any investigated CBs, so the amount of CB must be higher to reach the percolation limit and allow electrical conductivity. Increasing the CB ratio on 10 wt% results in an electrical conductivity for all three used CBs. As it can be seen, the maximum resistance varies significantly. At the maximal resistance, it is 390 kΩ for Vulcan XC-72, 230 kΩ for Ensaco 350G, and 90 kΩ for Ketjenblack EC-600JD. The initial resistances also follow this pattern. The differences occur due to the different particle sizes, BET surface areas, CB structures of the different CBs, and the interaction between the filler particles and the matrix material. As mentioned in Section 2.1, CBs with higher BET surface are often more likely to build complex structures, and therefore show lower electrical resistances. Ketjenblack EC-600JD, possessing the highest BET surface area (1400 [m²/g]), exhibits lower electrical resistances than the other two CBs. Ensaco 350G (770 [m²/g]) and Vulcan XC-72 (with the lowest BET surface area of 241 [m²/g]) confirm this trend, even though it is not as significant as the difference to Ketjenblack EC-600JD. 

When plotting the resistance over time, all three CBs at low CB ratios (10 wt%) exhibit a bimodal behavior. When the samples are stretched, the resistance initially increases, but then it decreases again before reaching the highest strain value. After reaching the maximum strain and relaxing again, this behavior is mirrored: the resistance increases during relaxation up to a second maximum, until it then decreases to the initial resistance value when the samples are completely relaxed, therefore forming a “dip” in the resistance–time plot. A detailed physical theory why this “dip” occurs is given in Section 3.3.

Increasing the CB ratio slightly leads, on one hand, to a lower overall resistance; on the other hand, the “dip” changes its shape. Firstly, the effect decreases and is less present, until it finally vanishes (at 15 wt% for Vulcan XC-72, 25 wt% for Ensaco 350G, and 15 wt% for Ketjenblack EC-600JD). Even when the bimodal behavior is not visible anymore, the curves show a shoulder, thus the resistance does not follow the strain directly. It is assumed that the “dip” is a strong expression of the observed phenomenon, which also leads to the shoulders at higher CB ratios. Ketjenblack EC-600JD (highest BET surface area) shows the least significant phenomenon. As the resistances of Ensaco 350G and Vulcan XC-72 (lowest BET surface area) are in the same order of magnitude, but the “dip” is more pronounced in Ensaco 350G, it cannot be said that the BET surface area is the dominant factor for the observed phenomenon. Further investigations on the structure of the CB agglomerates must be performed in future works. It is assumed that high-structure CBs such as the Ketjenblack EC-600JD are not as predisposed to this phenomenon as CBs, which form less connected structures.

Further increasing the CB ratio (> 20 wt% for Vulcan XC-72, > 25 wt% for Ensaco 350G and > 15 wt% for Ketjenblack EC-600JD) leads, on the one hand, to an even lower resistance, and, on the other hand, to a resistance behavior resembling the triangular strain–time curve (compare Figure 4). In this case, the “dip” cannot be observed anymore. At some point (> 25 wt% Vulcan XC-72, > 25 wt% Ensaco 350G, >10 wt% Ketjenblack EC-600JD), a further increase of the CB ratio no longer results in an automatic decrease in the resistance, but rather causes it to increase. Two effects contribute to this increase:

1. The amount of silicone is too low to hold the electrode together, resulting in a bad adhesion to the silicone membrane, worsening cohesion of the electrode itself and consequently easily rubbing off the electrode.
2. Due to the fabrication process, an increasing CB ratio also leads to an increasing amount of solvent in the electrode material to ensure screen-printability, leading to thinner electrodes and due to the manufacturing process to potentially more imperfections, as described in Section 2.2.

It should be noted at this point that an increasing CB usually leads to an increased electrode stiffness. When stretched, the force increases and the force–displacement hysteresis increases with a stiffer electrode. This would be unfavorable, e.g., for actuator applications but would not matter for DE sensors, which do not show large strains. In addition, stiffer electrodes are more prone to cracking and less flexible. This could explain the significant increase in the resistance when samples with a high CB ratio are stretched, in comparison to the samples with lower CB ratios. Nevertheless, no cracks could be observed when the samples examined in this work were stretched. Therefore, the significant increase in resistance is assumed to be a combination of different aspects: Thinner electrodes, imperfections in the film, as well as a lower cohesion of compositions with a higher CB ratios due to an insufficient amount of silicone matrix material. These points must be considered when designing DEs for specific applications. When designing material compositions for DEs, a rating of the importance for different characteristics must be carried out, e.g., if the resistance or the mechanical properties is more important for a specific application. In this paper, a fundamental analysis of the electrical resistance of screen-printed electrodes is conducted, therefore, no further investigations of the mechanical behavior are carried out in this work.

### 3.2. Influence of Silicone Matrices

In this section, the influence of the used silicone matrix material for the electrodes on the bimodal behavior is investigated. Other than Silgel 612 A/B, which is used as matrix material for the experiments in the previous section, Sylgard 182 A/B and NuSil R34-2168 are used for comparison. The same CB filler and the same filler ratio is used for the three silicones, thus excluding different influences of the CB on the results obtained in the following. The samples are prepared and stretched as described in Section 2.4. While in Section 3.1 only the resistance behavior was investigated, in this section, also, the stress–strain behavior is discussed, as it allows to further characterize the matrix material.

**Figure 6:**
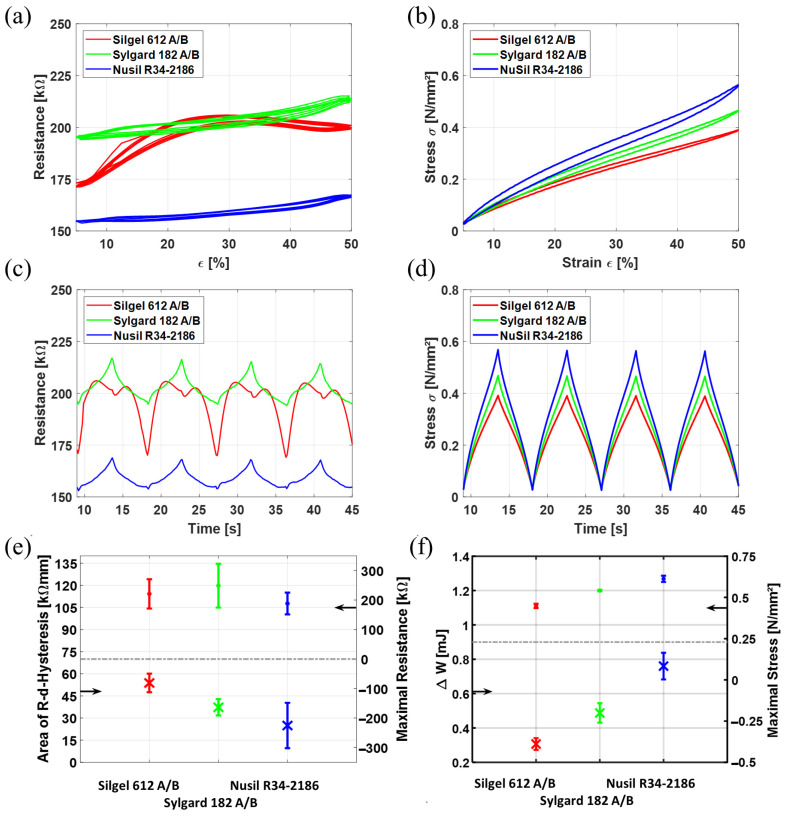
Influence of the used silicone on the electrical resistance and the mechanic behavior of the electrode: (**a**) Resistance–strain curves; (**b**) Stress–strain curves; (**c**) Resistance over time; (**d**) Stress over time; (**e**) Top: maximal resistance at ε = 50% = max, bottom: area of resistance–displacement–hysteresis: (**f**) Top: maximal stresses at ε = 50% = max, bottom: Work loss ΔW (corresponds to area of force–displacement hysteresis).

Figure 6 shows the results for these experiments. The left column depicts the results for the resistance measurements, while in the right column, the stress–strain behavior is shown. It should be noted that the electrodes are printed on a silicone membrane, which also has a stake of the measured force. The force values of the pure silicone membrane of the concrete samples can obviously not be measured. As for all samples the same silicone membrane of the same badge is used, it is assumed that the force stake of the silicone membrane is equal for all samples. In Addition, stress values for the samples do not start at 0 N/mm². The reason for this is given in Section 2.2: The silicone membranes are pre-stretched by 5% biaxially. Therefore, they show initial stresses when measured in the tensile tester. Figure 6b illustrates the influence of the different silicones on the stress values. Using Nusil R34-2186 as a matrix material results in the highest stresses. Sylgard 182 A/B shows a lower stress and Silgel 612 A/B exhibits the softest behavior. These curves represent the trend for all samples. The variations between samples of one sample type are resembled by the error bars in Figure 6f. Since Silgel 612 A/B shows the lowest stresses, it is assumed it has the highest polymer chain mobility as a matrix material, followed by Sylgard 182 A/B, and Nusil R34-2186. At this point it should be noted that stress values may be affected by additives and other substances in the purchased silicones used for manufacturing the electrodes. As all three materials are commercially available silicones, neither is it known which additional substances are in the silicones nor in which amount they are added, and finally how they are present in the final electrodes. The silicones are here, therefore, seen as a one-phase homogeneous material. 

Analyzing the stress–strain curves, it can be noted that with higher stress values, also the area of the corresponding hysteresis increases. To characterize the hysteresis, the mechanical work “W” performed by the system is calculated from the force–displacement curves corresponding to the shown stress–strain curves, according to:

W = ∫ F ds [J](1)

In Equation (1), F is the measured force in Newton and s is the displacement in meters. The hysteresis area is, therefore, the difference ΔW between the work W_stretch_, while stretching the samples, and the work W_unstretch_, while unloading the samples:

ΔW = W_stretch_ − W_unstretch_ [J] (2)

The unit of the work is Joule, and the hysteresis area of the force–displacement curve (which equals the work difference ΔW) is the energy, which is dissipated while stretching the sample for one cycle. As a lower hysteresis implies lower thermal losses, thus a higher energy efficiency, it is a good reference value for characterizing the stress–strain behavior of DEs. As the discussion in this paper is focused on the behavior of the resistance, no more detailed investigation on the hysteresis area is made. It should be noted that not only the energy efficiency increases with lower hysteresis areas in the stress–strain curves, but also a thinner hysteresis is better suited for the designing of DE actuators.

We compute the stress σ as:

σ = F/A [N/mm²](3)

In Equation (3) A is the cross-sectional area. The strain ε is defined as:

ε = (s/l_0_) × 100 [%] (4)

with l_0_ as the initial sample length. The work difference ΔW is directly proportional to the hysteresis area of the stress–strain curve. A trend in which the hysteresis area increases with increasing maximal stress can be clearly seen when the maximal stress values as well as the work difference ΔW are put against each other in Figure 6f.

In Figure 6a, one resistance–strain curve is displayed for each sample type. The absolute resistance values vary significantly between sample types. In Figure 6e also, the hysteresis of the resistance–displacement curves are displayed. Here, one can clearly see that the hysteresis for Silgel 612 A/B is higher than for Sylgard 182 A/B, and Nusil R34-2186 shows the lowest hysteresis. Interestingly, the order of the highest resistance–displacement hysteresis is opposite to the one of the force–displacement hysteresis. It seems that Silgel 612 A/B undergoes higher resistive changes during stretching, compared to the other two sample types, thus showing the highest resistance–displacement hysteresis. That allows us to conclude that higher polymer chain mobility, as is assumed for Silgel 612 A/B, allows more changes inside the electrode matrix. Figure 6c indicates that this assumption might be correct. There, the resistance over time curve is plotted. The “dip”, which can be observed for low CB ratios in the previous section, can only be seen when Silgel 612 A/B is used as a matrix material. Looking at the curves for Nusil R34-2186 and Sylgard 182 A/B, there, no “dip” can be observed. However, the shape of the curves also does not fit to the strain–time curve (Figure 4). Both exhibit shoulders, indicating that the phenomenon leading to the dip in the electrodes with Silgel 612 A/B as a matrix material also occurs in these samples, but with a weaker expression. The stronger the expression of the phenomenon is, the higher also is the resistance–displacement hysteresis. This would be unfortunate for DE resistive sensors, as with a wider hysteresis, no one-to-one transfer from one resistance to displacement value would be possible. One possible explanation why the bimodal behavior can only be seen when using Silgel 612 A/B may be since CB particles form agglomerates more easily in the more agile polymeric matrix, as when using a stiffer silicone matrix like Nusil R34-2168. This would result in bigger agglomerates, and therefore a worse dispersion of the CB particles. This theory should be further investigated in future work using image processing.

As the CB is responsible for the electrical conductivity of the electrodes, the change in electrical resistance during stretch is linked to the behavior of the CB in the polymeric matrix. Here, not only the state of CB particles, clusters, and agglomerates in the initial state is important. The resistance–stretch behavior is also connected to the polymer chain mobility. With higher polymer chain mobility, the CB move more easily inside the matrices while stretching and can therefore rearrange in different ways. A closer look at the possible explanation of the observed behavior is discussed in the next section.

### 3.3. Physical Explanation of CB Behavior in Silicone Matrices

Resulting from the observations gained from the previous sections, a physical explanation of the CB behavior inside the silicone matrix is given in the following. It represents a potential explanation for the resistance behavior when electrodes with different material compositions are stretched.

**Figure 7:**
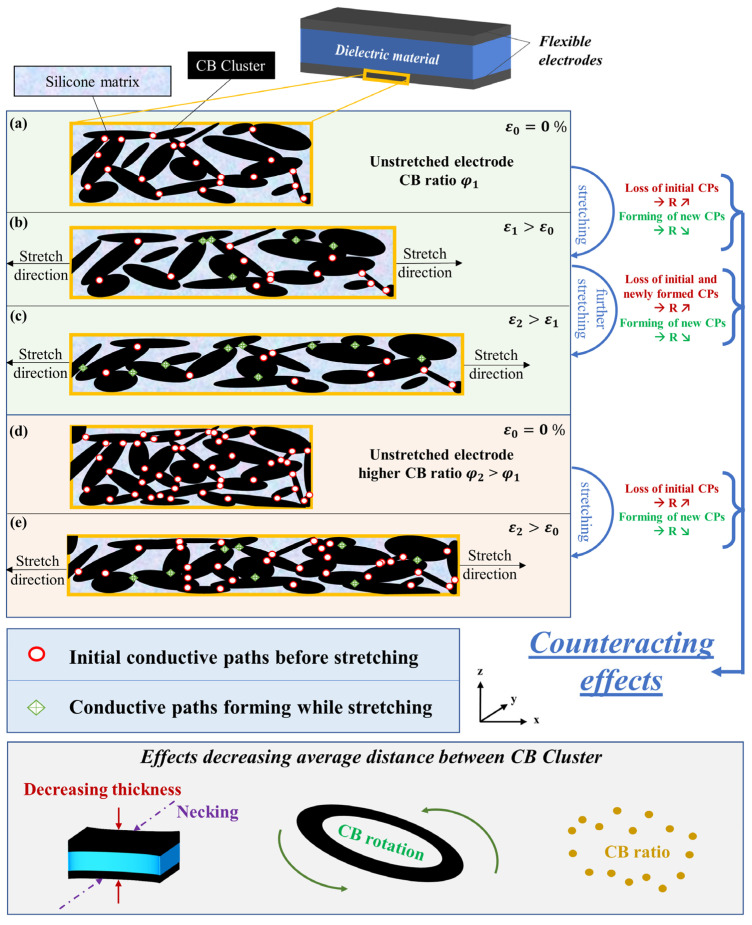
Schematic visualization of the CB behavior and the resulting conductive paths (CPs) while stretching to different strains ε_i_ (i = 0; 1; 2), simplified to a 2-dimensional view inside an electrode with CB as conductive particles and a polymeric matrix of a DE: (**a**) Unstretched electrode with a lower CB ratio φ_1_; (**b**) Stretched electrode with lower CB load φ_1_; (**c**) Further stretched electrode with lower CB load φ_1_; (**d**) Unstretched electrode with higher CB load φ_2_ > φ_1_; (**e**) Stretched electrode with higher CB load φ_2_ > φ_1_; Bottom: Schematic visualization of possible mechanisms, which decrease the average distance between CB clusters while stretching.

The CB are dispersed in the electrode material. After screen-printing and heat-curing the electrodes, the CB will be arranged in a statistical way, depending on their movement, structures, likelihood of cluster-forming and interactions with the matrix material. The cluster formation of CB particles is heavily dependent on their properties, as shown in the literature [19,35,43]. For the sake of simplicity, the CB clusters in this model are presented as ellipsoids with different lengths, widths, and orientations. In addition, the visualized model is presented as two-dimensional (thickness–length), although the electrode material is naturally a three-dimensional material and the discussed effects work in all dimensions. A scheme clarifying the physical explanation can be seen in Figure 7. In the cured but unstretched state (Figure 7a), a specific number of initial conductive paths (CPs) exist, presented as red circles. To create a CP, the CB clusters must either touch each other or be close to each other to allow tunneling effects. When two CB clusters are close enough, so a CP is already created due to tunneling effects, a decreasing distance leads to a lower potential barrier and, thus, decreasing the resistance of this specific conductive path, and therefore the electrical resistance of the whole system [37]. To make the basic idea clear and not to complicate the model, here, no differences are made if the potential barrier is high or low, we differentiate between if a CP exists or if there is no connection between two clusters. The number of CPs in the initial state depends on the used matrix material, the filler, additives, and on the manufacturing process. When stretching in x-direction (Figure 7b), the length of the electrode first increases, and the thickness decreases accordingly. Along the stretch direction, the average distance between the CB cluster increases, therefore some of the initial CPs will be lost, resulting in an increasing resistance [24,27]. On the other hand, due to the decreasing thickness, the average distance between the CB clusters in the thickness direction (z-direction) decreases [7,24]. The CB clusters change their orientation along the stretch direction, as well as complex clusters may change their shape [24]. As already mentioned, here, the CB clusters are assumed to be ellipsoidal. For less complex structures, which would be more spherical, the described effects would work similarly, whereas they would be less affected by change of orientation. Lastly, as we do not have an ideal DE with pure shear behavior, also necking perpendicular to the stretching and thickness direction occurs, decreasing the average distance between the clusters in y-direction. All three of these effects lead to a decreasing average distance between the CB clusters, and thus result in a forming of new CPs, represented as green diamonds crossed in Figure 7. These new CPs lead to a lower electrical resistance. If the loss of initial CPs is higher than the forming of new CPs, the resistance will increase while stretching. If the electrode is stretched further, exemplarily shown in Figure 7c, it is possible that even more CPs will be formed than the ones which are lost. For example, when most of the initial conductive paths in stretch direction are lost at a specific strain, resulting in an increasing electrical resistance, then the new forming of CPs in the other directions may become dominant. If this happens, the electrical resistance starts decreasing. It should be noted that, while stretching, the newly formed CPs can also be lost again due to slipping and shear effects. 

In conclusion, the behavior of the electrical resistance *R* depends on the summation of the number of lost CPs CP_loss_ and the number of newly formed CPs CP_form_:

- CP_loss_ > CP_form_ → R ↗
- CP_loss_ = CP_form_ → R →
- CP_loss_ < CP_form_ → R ↘

**Figure 8:**
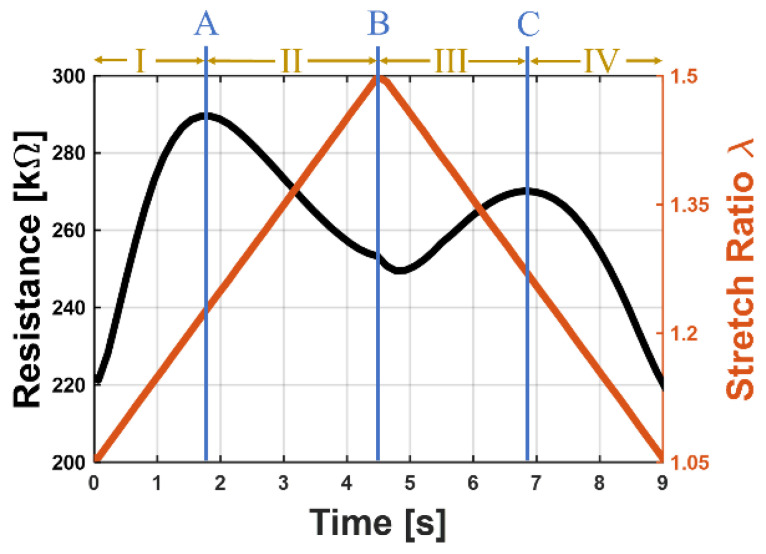
Distinguished regions and important points for the observed phenomenon based on an exemplary resistance-time behavior while one load-unload cycle: Region I: Increasing resistance with increasing elongation; Point A: First maximum of the resistance; Region II: Decreasing resistance while stretch ratio is still increasing; Point B: Maximal stretch ratio; Region III: The sample becomes unloaded, thus, the stretch ratio decreases and the electrical resistance increases; Point C: Second maximum of the resistance; Region IV: The electrical resistance decreases to its initial value when the sample is completely relaxed.

This means that, in the resistance behavior over time (e.g., Figure 8), four regions can be distinguished: In region I, the loss of conductive paths dominates, the resistance therefore increases. At higher stretches, fewer CPs are lost in stretch direction, as most of them are already broken. Additionally, more CPs are newly formed, leading to a slower increase of the resistance until point A, where the forming and losing of CPs are in an equilibrium. After this turning point, region II follows, where more CPs are formed than lost, resulting in a decreasing electrical resistance until the maximum stretch is reached (point B). In regions III and IV the samples are unloaded. The orientation now changes, and movements which happened during loading are now reversed. Therefore, the resistance first increases when most of the newly formed CPs are destroyed again, and after reaching an equilibrium (point C), the initial conductive paths in stretch direction which were destroyed at the beginning may form again. Therefore, regions III and IV are basically just region II and I mirrored, but with rate-dependent polymeric effects because of delay times of the polymeric matrix, therefore not showing a perfect symmetrical shape.

After providing an idea for the phenomenon, two questions remain open, i.e., why is the bimodal behavior only observable when using Silgel 612 A/B as a matrix material (compare Section 3.2), and why does the bimodal behavior only occur when using low CB ratios, and constantly becoming less intense when increasing the CB ratio (compare Section 3.1)?

Looking at the silicones as a matrix material, a softer polymer with a higher polymer chain mobility allows the movements, orientation changes, and shape changes of the CB clusters in an easier way than stiffer polymers with lower mobility. Therefore, this effect is predisposed to be more dominant in softer polymers and where new CPs may be formed easier. It should be noted, that, as mentioned in Section 2.1, the silicones used are commercially available ones. There might be other additives inside the material which are not known in this study and, therefore, the silicones were seen as a homogeneous phase. Nevertheless, if there are additives in the silicones present, they can alter the movement of the silicone polymer chains, and the movement of the CB. On one hand, they could blockade the movement of the CB clusters, if there are, e.g., stiff, and rigid fillers, on the other hand, they could improve the CB cluster movement, if they have a softening effect. It is, at this point, assumed that when fillers do have an influence on the movement of the polymer chains, these effects would work in the corresponding direction for the CBs as well, and, additionally, their effects would be included in the measured stress–strain curves of the polymers in Section 3.2.

In terms of the CB ratio, Figure 7 also shows the influence of a higher CB ratio. In this case, there are significantly more initial CPs in the unstretched state (Figure 7d), leading to an overall lower electrical resistance, which could also be seen in the results for the CB ratios in Section 3.1. When samples containing a lot of initial CPs are stretched (Figure 7e), the loss of CPs is more dominant. New paths will be formed, but as there are more CB clusters in the initial state which connect with each other, those new paths may not play a big role for the resistance, as clusters which come closer to each other may already be connected over another CP.

Now that all results are presented and the effects and interpretations for the observed behaviors are explained, one last question needs to be addressed: What do the different behaviors and phenomena observed in this work mean for the performance and designing of DEs? There is a tradeoff between the different characteristics of DEs depending on the desired application. Focusing on the results from this work, different effects can be observed, e.g., a narrow resistance–displacement hysteresis is accompanied by a wider force–displacement hysteresis when changing the silicone matrix material. Looking at different applications, different characteristics are important: For low frequency actuator applications, the electrode resistance is not as important as the mechanical behavior. The resistance must be in a suitable range and the electrode is not allowed to be damaged when stretched, therefore a rather low CB ratio (10 wt% to 20 wt%) and a softer silicone such as Silgel 612 A/B would be better suited. High frequency actuator applications, e.g., loudspeaker or haptic feedback systems, need a low resistance to allow these fast actuations. The lower resistance could be achieved at this point by a higher CB ratio or a stiffer silicone matrix material, which, however, entails a wider mechanical hysteresis. Looking at capacitive sensors, a monotonous and a hysteresis free resistance–strain behavior is favorable. Creating small sensors needs specifically designed electronics. Off-the-shelf standard capacitive measurement ICs (integrated circuits) work based on a constant serial resistance. In order for them to be applicable to DE sensors the resistance effects must be minimized. An unregular resistance behavior while being stretched can lead to a falsified measurement. In this case, a material composition with an electrical resistance following the strain–time curve is favorable, while the resistance changes should be as low as possible. The force–displacement hysteresis is in most of these applications no important factor, if the electrodes are not damaged and show repetitive behavior. Silgel 612 A/B with 25 wt% Ensaco 350G would be a suited material composition from the ones examined in this work. For the before mentioned resistive DE sensors, a monotonous and hysteresis free behavior is indispensable. A wide hysteresis as well as the bimodal behavior would be not suitable for such an application. For material compositions which show these behaviors, a resistance value cannot be clearly assigned to a strain value, which is an unavoidable exclusion criterion for them. Lastly, the mechanical hysteresis is not important, therefore a stiffer electrode containing of Nusil R34-2186 and a higher CB ratio like 30 wt% Vulcan XC-72 would be preferable. 

Summarized, for each application the appropriate material must be chosen. By investigating electrodes which are manufactured by screen-printing, it could be shown that the behavior of the electrical resistance is significantly influenceable by varying the electrode composition. The results obtained in this work give an overview and a theoretical explanation on how the material composition can be designed and what influence can be taken on the electrical resistance, depending on which behavior is desired for the planned application.

## 4. Conclusions

In this paper, the electrical resistance of a wide variety of PDMS-based CB electrodes for DE sensors and actuators is investigated regarding the material composition to determine preferred electrode compositions. The electrodes were prepared with the screen-printing method, which enables a repetitive process and therefore a good comparability for the investigated electrodes. The screen-printing method was also chosen because it is easily up-scalable for future mass production manufacturing The first part of the study focusses on different types of CBs using different CB to PDMS ratios to highlight the importance of CB content, in which all samples have the same silicone matrix material. In the second part of the study, different types of silicones are investigated. In this part, the CB to silicone ratio as well as the type of CB is kept constant to exclude the influence of different CBs while focusing on the influence of the used silicone matrix. Samples in both sections have been investigated by tensile test, where the electrical resistance is measured over elongation. For some compositions a bimodal behavior of the resistance while stretching was observed. For this phenomenon a potential explanation is also provided. It considers the behavior of the CB clusters inside the silicone matrix and is based on the changing ratio of losing and forming of conductive paths (CPs). While stretching, CPs in the stretch direction are destroyed, increasing the electrical resistance. On the other hand, due to necking, decreasing thickness and shape and orientation changes of the CB clusters, the average distance decreases perpendicular to the stretch direction. This leads to the forming of new CPs. Depending on the ratio of creating and destroying of CPs, different behaviors, e.g., a shoulder or a bimodal behavior in the resistance–time curves can be observed. The phenomenon leading to the bimodal behavior depends on different factors. First, the kind of CB used is important, as the structures different CBs form are different in size, shape, and complexity. Here, the Ketjenblack EC-600JD, as a CB which is known to build complex clusters and belongs to the ultra-conductive CB group, is less prone to this phenomenon than Ensaco 350G (extra-conductive CB) and Vulcan XC-72 (supra-conductive CB). A correlation of the BET-surface area and the expression of the phenomenon for the three used CBs has not been observed. Second, the filler ratio plays an important role. For increasing filler ratios, the overall electrical resistance in the initial state decreases, and the observed bimodal phenomenon decreases gradually while stretching the DEs. Other than CB, the used silicone material for the electrode is an important factor. Three different silicones, Silgel 612 A/B, Sylgard 182 A/B, and Nusil R34-2186 were used as matrix materials, where there the filler content is constant 16 wt%. The electrodes showed different results in stress–strain tests, as Silgel 612 A/B showed the softest behavior and smallest force-displacement-hysteresis area, followed by Sylgard 182 A/B and Nusil R34-2186. It is assumed that the lower stresses for Silgel 612 A/B result from higher polymer-chain mobility. Higher mobilities allow the CB clusters to move more easily inside the silicone matrix. Therefore, the named effects leading to the forming of new CPs are more likely to happen in a matrix with a higher polymer chain mobility, but also lead to a higher resistance–displacement hysteresis. The “dip” could only be observed when using Silgel 612 A/B, while for the others, one could observe shoulders. This indicates that the phenomenon also occurs when using stiffer silicones as matrix materials, but in a less significant expression. In summation, the electrical resistance and its behavior while stretching the electrodes strongly depends on the used materials, as well as on their relative amount of composition. Additionally, odd behaviors such as the bimodal behavior can be described to be dependent of the materials and their composition, allowing to alter those phenomena by changing the electrode composition. The electrical resistance of such systems is a complex topic with different importance for different DE applications, as discussed in detail in the previous section. Therefore, this paper highlights the need for an investigation of this property for applications, with an emphasis on the electrical resistance behavior. An experimental observation and validation of the assumptions and theories set up in this work needs to be carried out in future research.

## Figures and Tables

**Table 1:** Company and product names of the used silicones (left) and the used carbon blacks (right).

Silicones		Carbon Blacks	
Company	Product Name	Company	Product Name
Wacker Chemie AG (Munich, Germany)	Silgel 612 A/B	Akzonobel (Amsterdam, The Netherlands)	Ketjenblack EC-600JD
NuSil Technology (Carpenteria, CA, USA)	Nusil R34-2186	Imerys (Paris, France)	Ensaco 350G
Dow Chemical Company (Midland, MI, USA)	Sylgard 182 A/B	Cabot Corporation (Boston, MA, USA)	Vulcan XC-72

**Table 2:** BET surface area of the different CBs according to the manufacturer information.

Carbon Black	BET Surface Area [m²/g]
Ketjenblack EC-600JD	1400
Ensaco 350G	770
Vulcan XC-72	241

**Table 3:** Manufactured samples for first experimental section: Left: changing CB to silicone ratios; Right: used CB and the required amount of solvent for each electrode composition. Material compositions which showed no electrical conductivity are marked as n.e.c. = no electrical conductivity (could be achieved) while compositions which were not screen-printable anymore are marked as n.s.p. (not screen-printable).

Silicone to CB Ratio in Cured Electrodes		Prepared Samples with Different CB and the Required Amount of Solvent in the Electrode Printing Ink [wt%]		
Silgel 612 A/B [wt%]	CB [wt%]	Ketjenblack EC-600 JD	Ensaco 350G	Vulcan XC-72
95	5	58 (n.e.c.)	46 (n.e.c.)	33 (n.e.c.)
90	10	74	65	50
85	15	84	74	61
80	20	88	79	68
75	25	90	83	73
70	30	n.s.p.	86	76
65	35	n.s.p.	89	79
60	40	n.s.p.	n.s.p.	81

## Data Availability

The data presented in this study are available on request from the corresponding author. The data are not publicly available due to a close cooperation with mateligent GmbH.
